# CR-Mask RCNN: An Improved Mask RCNN Method for Airport Runway Detection and Segmentation in Remote Sensing Images

**DOI:** 10.3390/s25030657

**Published:** 2025-01-23

**Authors:** Meng Wan, Guannan Zhong, Qingshuang Wu, Xin Zhao, Yuqin Lin, Yida Lu

**Affiliations:** 1 School of Geography and Tourism, Anhui Normal University, Wuhu 241002, China; wanmeng@ahnu.edu.cn (M.W.); zgn390311648@163.com (G.Z.); zhaoxin123@ahnu.edu.cn (X.Z.); 2421011614@ahnu.edu.cn (Y.L.); yd@ahnu.edu.cn (Y.L.); 2 Heilongjiang Geomatics Center, Ministry of Natural Resources, Harbin 150001, China; 3 Anhui Provincial Engineering Technology Research Centre for Resource, Environment and Geographic Information, Wuhu 241003, China

**Keywords:** remotely sensed imagery, airport runway detection, improved Mask RCNN, rotated region generating network, attention mechanism

## Abstract

Airport runways, as the core part of airports, belong to vital national infrastructure, and the target detection and segmentation of airport runways in remote sensing images using deep learning methods have significant research value. Most of the existing airport target detection methods based on deep learning rely on horizontal bounding boxes for localization, which often contain irrelevant background information. Moreover, when detecting multiple intersecting airport runways in a single remote sensing image, issues such as false positives and false negatives are apt to occur. To address these challenges, this study proposes an end-to-end remote sensing image airport runway detection and segmentation method based on an improved Mask RCNN (CR-Mask RCNN). The proposed method uses a rotated region generation network instead of a non-rotated region generation network, allowing it to generate rotated bounding boxes that fit the shape of the airport runway more closely, thus avoiding the interference of a large amount of invalid background information brought about by horizontal bounding boxes. Furthermore, the method incorporates an attention mechanism into the backbone feature extraction network to allocate attention to different airport runway feature map scales, which enhances the extraction of local feature information, captures detailed information more effectively, and reduces issues of false positives and false negatives when detecting airport runway targets. The results indicate that, when comparing horizontal bounding boxes with rotated bounding boxes for detecting and segmenting airport runways, the latter are more precise for complex backgrounds. Furthermore, incorporating an attention mechanism enhances the accuracy of airport runway recognition, making it highly effective and practical.

## 1. Introduction

Target detection in remote sensing images is a current research hotspot in the field of image processing. Airports, as crucial national infrastructure, play an essential role in aviation transportation, supporting key tasks such as the take-off and landing of aircraft [1]. However, due to the fact that airports have varied shapes and complicated backgrounds, and are sensitive to interference from a variety of environmental features such as roads and residential areas around the airports, accurate and real-time airport detection is still problematic and challenging.

The airport runway is the core part of the airport. Its detection and identification in remote sensing images can provide a reference for the understanding and application of the whole image. As it is a good point of information extraction, it is customary to detect airports through the identification of airport runways [2]. With the construction, renovation, and expansion of airports in various countries, airport runways have undergone significant changes. The rapid and efficient detection and extraction of runways have become a key area of research, especially in the airport monitoring system, where the application of airport runway detection technology can effectively identify obstacles and damage on the runway to ensure the safety of flights. In addition, in emergency management scenarios, the use of airport runway detection can also help to quickly assess the damage to an airport after a disaster and speed up rescue and recovery work. Therefore, airport runway inspection technology not only plays an important role in routine monitoring, but also has importance in post-disaster emergency responses.

Traditional target detection methods for airport runways from remote sensing images are mainly based on visual saliency methods. The visual saliency method mainly utilizes runway flatness, surface uniformity, herringbone markings, and other aspects to extract underlying features, such as linear features, texture features, and shape features, to locate and extract the runway [3,4]. The main issue with this method is its dependence on custom thresholds to detect features. These thresholds are typically established based on particular datasets or conditions, lacking the necessary flexibility and adaptability. As a result, when faced with environments and conditions that differ from the original training or threshold settings, these thresholds may fail to accurately distinguish between runway and non-runway features, increasing the risk of false positives. Furthermore, traditional airport runway target detection systems are generally constrained by their feature expression capabilities and computing efficiency, making them inadequate for complicated scenarios.

With the rapid advancement of deep learning technology, an increasing number of researchers have applied deep learning methods to many challenging tasks such as image classification [5], target recognition [6], image segmentation and detection [7], and so on, achieving promising results. Consequently, based on deep learning, certain researchers have delved into investigating target detection for airports and airport runways in remote sensing images.

For instance, Yin [8] utilized the Faster RCNN algorithm to detect airports in large-scale remote sensing images and implemented an improved multi-task loss function to enhance the precision of airport detection. Xu [9] applied the Faster RCNN algorithm as the foundational framework and introduced migration learning to tackle issues such as limited airport data. The approach involved transferring knowledge from training on natural images to enhance airport detection in remote sensing images, mitigating overfitting caused by data scarcity. Chen [10] introduced a rapid and automatic airport detection method based on convolutional neural networks. The system employs an enhanced Faster RCNN algorithm to effectively identify airports, particularly handling the distinctive long linear geometry of airport runways. Khelifi [11] evaluated the performance of Faster region-based convolutional neural networks using advanced backbone architectures—namely, ResNet-101 and ResNet-X152—for the detection of airport runways. Li [12] introduced a lightweight remote sensing rotated target detection model for YOLOv5 to address the challenge of detecting rotated objects with various angles, such as airplanes. The model converted the angular regression problem into a classification problem such that the model predicts one more item of angle information for a rotated horizontal bounding box to enclose rotated objects with arbitrary angles. Ji [13] introduced a rapid and automatic airport detection method based on convolutional neural networks by means of YOLOv3’s dual-scale runway detector for the swift and automatic verification of the spatial location of an airport runway, then used ResNet-101’s refinement classifier to enhance the precision of the initial detection results.

Existing airport runway target recognition techniques based on deep learning are mostly categorized into two groups. One is to convert the detection into a regression problem, represented by YOLO networks, which enhance the detection speed by directly predicting the bounding box. However, the YOLO network’s grid mapping of the original image may lead to imprecise target localization, resulting in low accuracy and subpar detection performance. The other is region-based target detection, such as RCNN, Fast RCNN, and Faster RCNN [14,15,16]. Compared with regression methods, these algorithms adopt a two-stage detection strategy. Initially, the region proposal network (RPN) generates horizontal bounding boxes to locate the target. Then, these bounding boxes are classified and refined through bounding box regression, thereby improving the accuracy of the detection results.

However, most two-stage target detection algorithms are designed for natural scenes and generally generate horizontal bounding boxes to locate the target. When detecting airport runways, the outermost linear shape of the airport runway is used as the selection box. However, this bounding box covers a lot of background information, reducing the detection accuracy for long, narrow, and rotated targets. In addition, a single remote sensing image can easily contain multiple intersecting airport runway targets, and the airport runway is closely connected, making it easy to lose feature information, resulting in low detection accuracy and a high false negative rate.

Compared to Faster RCNN, Mask RCNN [17] performs object detection and bounding box regression and enables pixel-level object segmentation. This feature allows for higher detection accuracy, especially when handling objects with complex shapes and irregular contours. Therefore, to address the challenges in airport runway detection, this study proposes an improved Mask RCNN method for detecting and segmenting airport runways, aimed at more accurately identifying and extracting airport runway contour information.

Firstly, this study selected images containing airport runways from the DIOR dataset [18] and annotated the filtered runway images to construct a sample dataset suitable for airport runway detection. Based on this airport runway dataset, to effectively handle the detection of airport runways in arbitrary orientations, the rotated region proposal network (RRPN) was adopted to replace the standard RPN. This approach generates rotated bounding boxes that better fit the shape of airport runways, thereby reducing the interference caused by the large amount of irrelevant background information associated with horizontal bounding boxes. Secondly, to address the problem of false negatives and false positives that easily arise in the detection of targets containing multiple intersecting airport runways in a single image, this study further incorporates an attention mechanism into the backbone feature extraction network. This addition reduces the path length during the information transfer process in the deep network structure and enhances the extraction of feature information to capture detailed information more effectively, thus mitigating issues that may lead to the loss of feature information and lowering the possibility of false negatives and false positives. Finally, the proposed CR-Mask RCNN method demonstrates significant advantages through experimental application and comparative analysis. The main contributions of this study are as follows:

(1) A method for end-to-end airport runway detection and segmentation in remote sensing images based on an improved Mask RCNN is proposed to enhance the capability of feature extraction for airport runways.
(2) To address the detection of airport runways, which are long, narrow, and rotational targets with variable orientations, this study uses RRPN to replace the traditional RPN, in order to avoid the interference of irrelevant background information caused by horizontal bounding boxes. Meanwhile, when extracting the region of interest (ROI), the RROI Align layer is used to replace the ROI Align layer, ensuring effective feature alignment under rotation and maintaining the spatial consistency of airport runways on the feature map, thus accurately cropping the features that match the target region. This method significantly improves the accuracy of the detection and segmentation of airport runways; in particular, it shows more pronounced effects in addressing the issue of false negatives of airport runways in large-scale images.
(3) An attention mechanism is incorporated into the backbone feature extraction network, which enhances the response of the feature extractor to airport runway targets and suppresses background noise. This improvement further increases the accuracy of airport runway detection, significantly enhances the precision of airport runway segmentation, and reduces the occurrence of false positives and false negatives in airport runway detection. In particular, when handling multiple intersecting airport runways in a single remote sensing image, it effectively reduces the probability of false positives and false negatives.

## 2. Methods

### 2.1. Base Network Model

**Figure 1:**
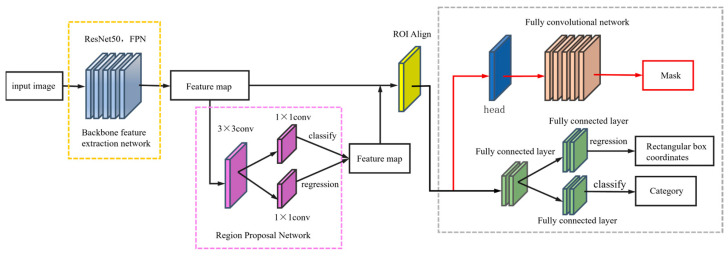
Structure diagram of Mask RCNN network model.

While the deep learning convolutional neural network plays an important role in the field of target detection, the target detection model is also constantly optimized and updated. He Kaiming et al. [17] proposed an improved Mask RCNN network model based on Faster RCNN (Figure 1), which adds the FCN semantic segmentation module on top of Faster RCNN and simultaneously achieves the target. The Mask RCNN also introduces a feature pyramid network (FPN), which makes full use of the multilayer feature maps from low to high resolution to improve target detection and segmentation accuracy.

The model is divided into three main parts. Firstly, a bottom-up, top-down and horizontal connectivity network is combined in the backbone feature extraction network to obtain feature maps at different scales and fuse them, thus equipping the network with high-resolution shallow features and richer deep feature information. Secondly, the RPN is used to extract bounding boxes for target features. The RPN generates a series of bounding boxes by sliding a small window (typically a 3 × 3 filter) over the feature maps in the backbone feature extraction network. The feature map within each sliding window is then mapped to a low-dimensional feature vector through a convolutional layer, followed by score calculation, thereby providing high-precision target bounding boxes.

Finally, when extracting the ROI, the Mask RCNN network model performs another important optimization by replacing the ROI Pooling layer with an ROI Align layer, reducing the mapping relationship error between the original image and the extracted feature maps. ROI Align uses bilinear interpolation to calculate the coordinate values of the sampled points in each grid after segmentation and then pools their maximum value. The coordinates of the bounding boxes obtained in this way are almost the same as the original coordinates, which drastically reduces the mapping error and, thus, improves the detection accuracy of the network.

### 2.2. Improvement Strategy of Mask RCNN Based on Airport Runways

This study improves the Mask RCNN network model based on the current problems of deep learning in airport runways. Firstly, a rotated region generating network (RRPN) is used instead of the traditional region generating network (RPN) [19,20] to generate rotated bounding boxes that fit the shape of the airport runway more closely. Secondly, ROI Align is used to optimize the ROI Align layer of the Mask RCNN model to accurately crop out the features in the feature map that match the airport runway area, ensuring that no important information about the airport runway is lost. Finally, an attention mechanism is further introduced into the backbone feature extraction network to reduce the false negatives and false positives in airport runway detection.

#### 2.2.1. Replace the RPN Network with the RRPN Network

**Figure 2:**
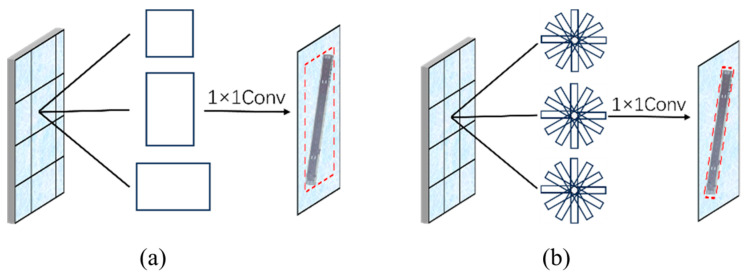
Two types of region bounding boxes. (**a**) Horizontal bounding box; (**b**) rotated bounding box.

The traditional Mask RCNN network uses RPN to generate horizontal bounding boxes, as shown in Figure 2b, which is suitable for horizontal target detection but has a limited effect in dealing with rotated targets. Airport runways usually present long and narrow shape features with multiple rotation angles, and the horizontal bounding boxes generated by RPN will contain a large amount of background information, thus affecting the detection accuracy. Therefore, in the region proposal generation network of the Mask RCNN network model, this study replaces the RPN with RRPN, whose bounding box generation mechanism is controlled by scale, aspect ratio, and angle, and can generate rectangular boxes in any direction [21,22,23], as shown in Figure 2b.

**Figure 3:**
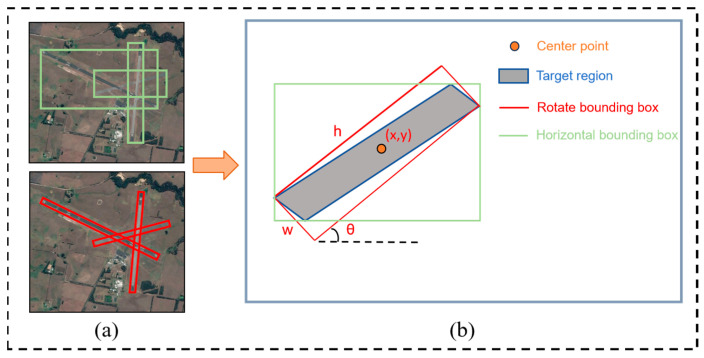
Schematic diagram of horizontal and rotated bounding boxes. (**a**) Bounding box diagram; (**b**) bounding box parameter representation.

Assuming that the size of the remote sensing image of the airport runway is $I_{h}\times I_{w}$, after adding the angle parameter, the airport runway rotation bounding box can be expressed as (x, y, h, w, θ). Figure 3a illustrates the horizontal bounding box (top) and the rotated bounding box (bottom) generated for the airport runway by the RPN and RRPN networks in the remote sensing image. Figure 3b is a schematic diagram illustrating the parameters of the rotated bounding box. Here, (x, y) represents the center coordinates of the bounding box, h and w are the height and width of the bounding box, respectively, and θ is the angle between the X-positive axis and the long side of the bounding box, i.e., the rotation angle [24].

Considering that the model is able to identify the direction and its opposite direction of the target, in this study, we adopt a special treatment to optimize the setting of the angle parameter θ in the rotated bounding box model. We fix the value of θ so that it covers half of the angular space and computationally find a unique integer k such that the result of θ+kπ falls within half of the angular interval, thus maintaining the consistency of θ and reducing redundant direction detection. When θ+kπ exceeds the specified interval, we update it to θ+kπ. This approach simplifies the computation process and improves the accuracy of target detection, which is especially suitable for the airport runway detection scenario in this paper. During the training process, we also perform data enhancement by rotating an angle α(α ∈ [0, 2*π*)) around the center of the image, when the orientation of the bounding box is θ=θ+α+kπ. The bounding box center is calculated as follows:

(1) $$\left[\begin{array}{c} x^{\prime }\\ y^{\prime }\\ 1 \end{array}\right]=T\left(\frac{I_{W}}{2},\frac{I_{H}}{2}\right)R\left(\alpha \right)T\left(-\frac{I_{W}}{2},-\frac{I_{H}}{2}\right)\left[\begin{array}{c} x\\ y\\ 1 \end{array}\right]$$

where T and R are the translation and rotation matrices, respectively.

(2) $$T\left(\delta_{x},\delta_{x}\right)=\left[\begin{array}{ccc} 1 & 0 & \delta_{x}\\ 0 & 1 & \delta_{x}\\ 0 & 0 & 1 \end{array}\right]$$

(3) $$R\left(\alpha \right)=\left[\begin{array}{ccc} \cos\alpha & \sin\alpha & 0\\ -\sin\alpha & \cos\alpha & 0\\ 0 & 0 & 1 \end{array}\right]$$

In the optimization process of rotated bounding boxes, there is a trade-off between accuracy and efficiency. The accuracy and consistency of the model can be significantly improved by adjusting the angular parameters so that they fall within the effective range, especially when dealing with rotated targets. However, this adjustment requires additional matrix transformations to realize, increasing the computational complexity. Therefore, while the accuracy is improved, the computational overhead also increases, requiring appropriate trade-offs in the application.

As there are not only scale and positional differences but also angular differences between the generated rotated bounding box and the real box, the original border regression method (correcting only the position width and height) is unable to carry out the angular regression. Thus, using the rotated bounding box representation in the RRPN network to correct the borders, bounding box regression is only performed on the positive samples and they must satisfy the following conditions:

(1) The Intersection Over Union (IOU) ratio between the bounding box and the sample true label is greater than 0.7.
(2) The angle difference between the bounding box and the ground truth label is less than 15°.

**Figure 4:**
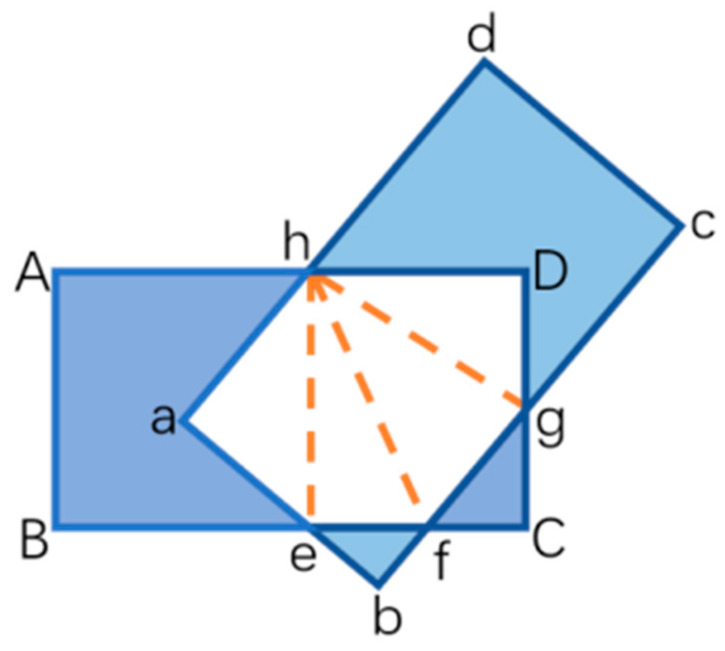
IOU calculation diagram.

Negative samples are determined when the IOU between the bounding box and the real sample label is less than 0.3 and when the IOU between the bounding box and the real label of the sample is greater than 0.7, but the angle between the two boxes is greater than 15°. Bounding boxes that do not satisfy both positive and negative samples are discarded and are not involved in the training and learning of the network model. The calculation method for the IOU between the bounding box and the real label of the sample is shown schematically in Figure 4, and the specific steps of the calculation are shown in Table 1.

#### 2.2.2. Optimizing the ROI Align Layer

The ROI Align layer in the Mask RCNN network model target region alignment network is a regular rectangle-based method for the accurate cropping and feature alignment of regions of interest. However, for the rotated bounding region facing the target of an arbitrarily orientated airport runway generated by the RRPN in this study, the traditional ROI Align may not be able to capture the accurate information of an airport runway with a rotation angle. Therefore, this study adopts RROI Align to replace the ROI Align layer [25,26]. RROI Align can align features efficiently under rotation to maintain the spatial consistency of the airport runway on the feature map and accurately crop out the features in the feature map that match the region to ensure that no vital information about the airport runway is lost.

**Figure 5:**
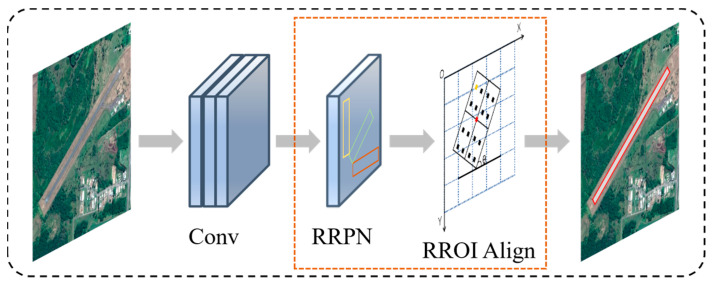
RROI Align calculation flowchart.

ROI Align uses bilinear interpolation to process the feature maps where the bounding boxes are located to avoid inaccurate features caused by the boundary. The sampling points selected by ROI Align for bilinear interpolation are coordinate-shifted, and the formula for calculating the coordinate-shifting process is shown in Equation (4), so that the rotated boxes can be mapped accurately in the corresponding feature maps through the ROI Align layer. The RROI Align layer can map these rotated bounding boxes accurately on the corresponding feature maps.

(4) $$\begin{array}{c} x=yy*sin\theta +xx*cos\theta +{\mathrm{center}}_{w}\\ y=yy*cos\theta -xx*sin\theta +{\mathrm{center}}_{h} \end{array}$$

where the blue dashed line represents the airport runway feature map and the black solid line represents the sampled region of interest; xx and yy, respectively, denote the coordinates of the sampled points in the feature map; ${\mathrm{center}}_{w}$ and ${\mathrm{center}}_{h}$, respectively, denote the coordinates of the center point of the feature map (red dot); and θ denotes the current rotation angle, as shown in Figure 5.

In the loss function calculation part of the network model, the loss function of RPN consists of classification loss and regression loss. The classification loss function usually uses cross-entropy loss and the regression loss function usually uses smooth L1 loss. However, the RPN regression loss function only contains the position regression loss. As this study adopts RRPN to replace RPN in the Mask RCNN network model, it is necessary to add the angular regression loss to the regression loss function, and the calculation formula is shown in Equation (5).

(5) $$L\left(p,l,v^{*},v\right)=L_{cls}\left(p,l\right)+\lambda lL_{reg}\left(v^{*},v\right)$$

where p denotes the confidence level of the category; l denotes the positive and negative sample labels for the rotated bounding box (when l is 1, it indicates a positive sample; when l is 0, it denotes a negative sample); $v^{*}$ denotes the parameterized coordinates of the bounding box of the rotated bounding box and the true sample; and v denotes the parameterized coordinates of the bounding box of the rotated bounding box with respect to the target prediction result.

#### 2.2.3. Integrate the Attention Mechanism into the Backbone Feature Extraction Network

The complex background information in remote sensing images easily interferes with the network when extracting the target, and a single image often contains multiple closely connected airport runway targets, which are prone to losing feature information, resulting in low detection accuracy and a high false negative rate. For this reason, the attention mechanism [27] can help the network to focus on the target area and improve the amount of target feature information.

**Figure 6:**
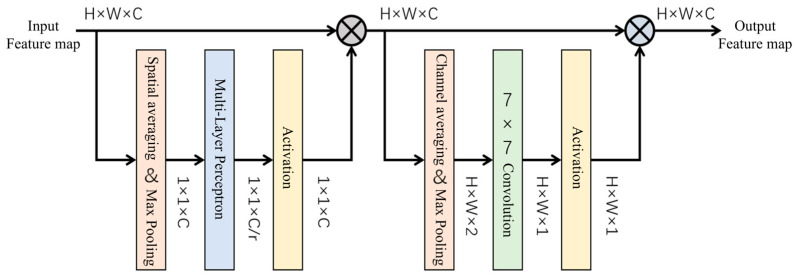
CBAM structural diagram.

CBAM (Convolution Block Attention Module) is a lightweight attention module that combines spatial and channel hybrid attention mechanisms, and its structure is shown in Figure 6. By generating the attention graph in channel and spatial dimensions, it is able to enhance the network’s ability to perceive information. Where channel attention assigns weights according to feature importance and spatial attention focuses on the foreground region, combining these two mechanisms, CBAM helps the network to extract important feature information more efficiently and improve detection accuracy [28,29].

The channel attention module first performs global average pooling (AvgPool) and max pooling (MaxPool) on the input feature map. It then obtains the descriptive features $F_{c\_avg}$ and $F_{c\_max}$, respectively. Then, these two features are processed by the shared multilayer perceptron (MLP) for feature summation operation and by the Sigmoid function to obtain $M_{c}\left(F\right)$. Finally, the channel attention weight $M_{c}\left(F\right)$ is multiplied with the input feature map F to obtain the channel attention feature map $F^{\prime }$, as shown in Equations (6) and (7).

(6) $$M_{c}\left(F\right)=\sigma (MLP(F_{c\_avg})+MLP(F_{c\_max}))$$

(7) $$F^{\prime }=M_{c}(F)⊗F$$

The spatial attention module then takes the input feature map. Average pooling and maximum pooling operations are performed to obtain the respective descriptive features. These two features are then aggregated by horizontal splicing and passed into a 7 × 7 convolutional kernel for operation (*f*^7×7^). Next, the obtained results are subjected to a Sigmoid (σ) operation. Finally, by combining the spatial weighting coefficients with the input feature map multiplication, the hybrid attention feature map is obtained, as shown in Equations (8) and (9).

(8) $$M_{s}\left(F^{\prime }\right)=\sigma (f^{7\times 7}([F_{s\_avg};F_{s\_max}]))$$

(9) $$F^{\prime \prime }=M_{s}(F^{\prime })⊗F^{\prime }$$

**Figure 7:**
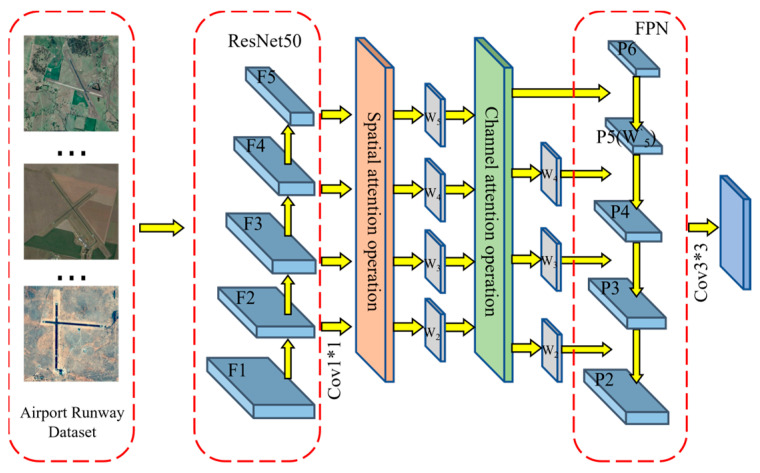
Structural diagram of backbone feature extraction network with integrated attention mechanism.

After the model is optimized using RRPN and RROI Align, it can better cope with the detection of rotated targets. Based on this, this study applies the attention mechanism to the Mask RCNN network model backbone feature extraction network, thus helping the network dynamically allocate attention to the airport runway feature maps at different scales. In this way, the lower-resolution but semantically information-rich airport runway feature maps can receive more attention, while the higher-resolution airport runway feature maps can be appropriately modulated to better capture the detail information, thus improving the perceptual ability and accuracy of the model. The structural diagram of the backbone feature extraction network introducing the attention mechanism is shown in Figure 7.

## 3. Experiments and Analyses

### 3.1. Airport Runway Experimental Dataset

Numerous deep learning publicly available remote sensing imagery datasets, such as the DOTA and DIOR datasets, have emerged to meet the needs of scientific research and network modeling competitions, and these datasets contain a large number of class annotations of common feature targets such as vehicles, aircraft, and ballparks [18,30]. However, relevant publicly available datasets are relatively scarce due to relatively limited research on airport runways. Therefore, to meet the research needs in this paper, we chose to adopt the existing public remote sensing image datasets, extract the images containing airport runways and annotate them, and construct the remote sensing image dataset of airport runways.

**Figure 8:**
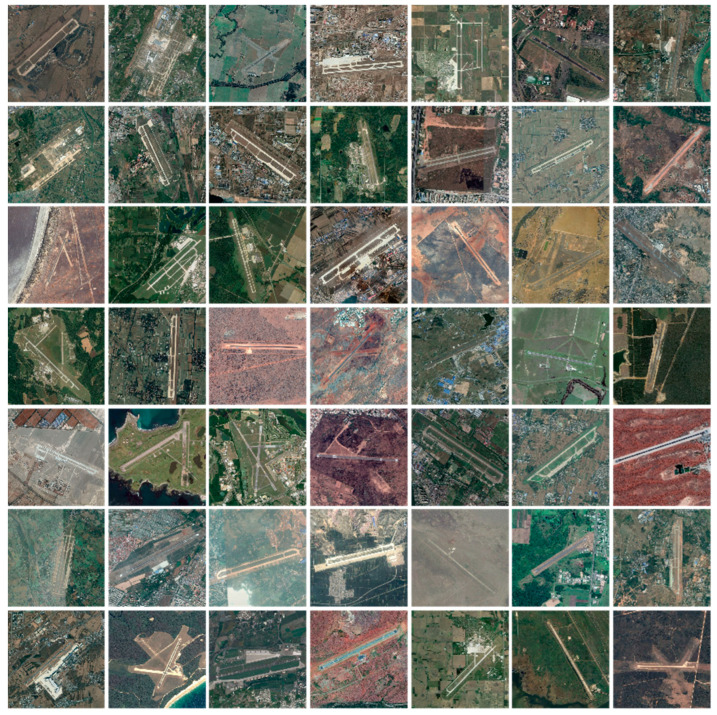
Airport runway remote sensing image dataset filtered from DIOR dataset.

In this study, by comparing the quantity and quality of airport runway images contained between different datasets, the DIOR dataset was chosen as the source of remote sensing images of airport runways. The DIOR remote sensing image dataset was proposed by Northwestern Polytechnical University in 2018 with the production of remote sensing image data in Google Earth, which contains a large range of such data. It is publicly available, which is convenient for researchers to use in different fields. In addition, the DIOR dataset contains 23,463 remote sensing images and 190,288 instances, covering a wide range of feature types and environments. In particular, it provides a large number of airport runway images, which can provide a rich database for airport runway detection and segmentation tasks. Compared with other datasets, DIOR offers images with different resolutions from 0.5 to 30 m, which helps to achieve good generalization ability in different scenarios, thus ensuring that the proposed method has a wide range of applicability in the real world. In the research process, the DIOR dataset airport runway remote sensing image data were first screened. Then, the obtained images were manually contrasted and checked to exclude unusable images such as category errors. Finally, 1263 basic remote sensing images containing airport runways were screened, as shown in Figure 8.

**Figure 9:**
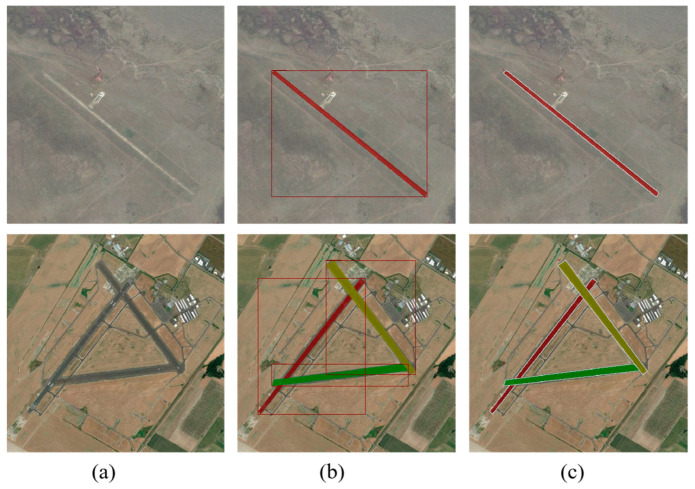
Two types of annotations in airport runway dataset. (**a**) Original image; (**b**) horizontal box annotation, the red box indicates the detection annotation and the internal color indicates the segmentation annotation; (**c**) rotated box annotation, the white box indicates the detection annotation and the internal color indicates the segmentation annotation.

Subsequently, 1263 remote sensing images of airport runways were annotated, as shown in Figure 9. This study employed two annotation methods based on the horizontal and rotated bounding boxes generated by the RPN and the RRPN, respectively, to cater to the requirements of horizontal and rotated airport runway detection methods. The original airport runway images were annotated with both horizontal and rotated bounding boxes. The Labelme annotation tool was used for this purpose, and the annotations were then converted into the COCO dataset format utilized by the Mask RCNN model [31]. Additionally, the dataset was split into training and validation sets in a 7:3 ratio, with the training set used for model parameter training and the validation set for performance evaluation and hyperparameter tuning.

### 3.2. Evaluation Indicators

In the target detection task of deep learning, accuracy, i.e., the AP value, is generally used as an evaluation metric for the training of the network model. The AP value needs to be calculated by Precision, which is statistically computed from the prediction results of real samples by the network model, as well as Recall.

As the target detection carried out in this study is a single-category detection, the positive samples are airport runways and the negative samples are non-airport runways. Therefore, the detection results can be counted as a dichotomy (i.e., the relationship matrix between the real samples and the predicted results), as shown in Table 2.

The method of judgement for the predicted classification results is statistically determined by calculating the results of the IOU. The IOU represents the proportion of intersections and concatenations between the predicted boxes and the real labeled boxes. The calculation formula is shown in Equation (10).

(10) $$IOU=\frac{A\cap B}{A\cup B}$$

After the above calculation statistics, the Precision and Recall calculations are performed.

The formula for Precision is shown in Equation (11):

(11) $$Precision=\frac{TP}{TP+FP}$$

The formula for Recall is shown in Equation (12):

(12) $$Recall=\frac{TP}{TP+FN}$$

The F1 Score is the harmonic mean of Precision and Recall, used to measure the overall performance of a model in object detection or segmentation tasks. Its calculation formula is shown in Equation (13):

(13) $$F1=2\times \frac{Precision\times Recall}{Precision\times Recall}$$

In object detection and segmentation tasks, relying solely on a single evaluation metric may not comprehensively assess the network model. The combination of Precision and Recall to compute the average precision (AP) is currently the most commonly used and reliable evaluation method in object detection. The calculation of AP involves determining the area under the curve generated by the Precision and Recall metrics, which are enclosed by the X and Y axes. The formula for this calculation is presented in Equation (14).

(14) $$AP=\int_{0}^{1}P\left(R\right)dR$$

### 3.3. Experimental Environment and Setup

The experiments in this study were conducted on a platform with a 7-core ubuntu system, a GeForce RTX3060 graphics card with 12 G video memory, and a Xeon(R) E5-2680 v4 processor. The hyperparameters used for the training are set as follows: the number of iterations was 200, the initial learning rate was set to 0.002, the learning rate was decayed at the 100th and 150th times with a decay multiplier of 0.1, the SGD stochastic gradient descent method was chosen to optimize the model parameters, and the impulse unit momentum was set to 0.9.

The choice of learning rate is a key factor affecting the model training speed and convergence performance. A lower learning rate can avoid the training instability problem caused by too large a step size at the early stage of training. The decay rate was set to 0.1 to ensure that, in the later stages of training, the decrease in the learning rate can refine the parameters of the model more effectively, thus improving the convergence and accuracy of the model. The decay of the learning rate at the 100th and 150th iterations was adjusted according to the training progress; using a larger learning rate at the initial stage helps to converge faster, while gradually decreasing the learning rate at the later stage helps to improve the accuracy and stability of the training, and to avoid oscillations during the training process. The SGD stochastic gradient descent method was chosen to optimize the model parameters, and the momentum term (momentum) of the optimization process was set to 0.9, which helps to accelerate the convergence speed of the network and, at the same time, avoids the local optimal solution that may appear during the training process, and improves the generalization ability of the overall model.

In addition, as the length and width of the airport runway is relatively large, to ensure the ratio of the bounding box conforms well to the airport runway, by counting all the images in the airport runway dataset and calculating the aspect ratio of the rotated bounding box generated by its annotation, we obtain the results shown in Table 3. That is, the aspect ratios of most of the runways are distributed between 1:20 and 1:60, so the aspect ratios of the bounding box were set to {1:25, 1:35, 1:45, 1:55, 55:1, 45:1, 35:1, 25:1}.

For the setting of the angular parameter θ of the rotated bounding box, in general, smaller angular intervals result in improved detection accuracy, but this often leads to an increase in computational cost, which prolongs the processing time and increases the memory requirement, and additionally too small an angular interval may lead to a lack of GPU memory, which triggers an interruption of the training process. Considering the limited GPU resources in this study, in order to find the optimal balance between improving the accuracy of the network model and maintaining the computational efficiency, as well as to ensure the feasibility of the experiment, in this study, 15° was chosen as the angular interval for rotated bounding boxes. With this setting, both the detection accuracy can be effectively optimized and the high accuracy of the model can be guaranteed with a small loss of accuracy, while achieving a good balance between computational efficiency and accuracy, thus significantly reducing the computational complexity. Therefore, the 15° interval setting not only improves the detection performance, but also ensures that the method is efficient, scalable, and operable in this study, and can better adapt to the constraints of hardware resources.

Non-maximum suppression (NMS) of rotated bounding box targets is particularly critical in the application of rotated bounding box target detection, especially when faced with airport runway targets with long and narrow shapes and arbitrary orientations. IOU, as a decision criterion, is sensitive to the rotation angle and the aspect ratio, which leads to large discrepancies under slight variations. As a result, this sensitivity may lead to too many bounding boxes in the results or incorrect suppression of the correct boxes during NMS, thus affecting the accuracy of the network model. Therefore, the choice of using the Skew-NMS method and the continuous debugging of the network model parameters finally led to the determination of suppressing the bounding boxes in the following two ranges:

(1) When the IOU with the bounding box that has the highest confidence level is greater than 0.5.
(2) When the IOU between two bounding boxes is greater than 0.3 and less than 0.5 while their angle difference is less than 15°.

### 3.4. Analysis of Experimental Results

Based on the airport runway dataset constructed in this study, we performed both qualitative and quantitative comparisons between the proposed method and the original Mask RCNN model (the rotated Mask RCNN, with RRPN replacing the RPN, is denoted as R-Mask RCNN, and the rotated Mask RCNN with an attention mechanism is denoted as CR-Mask RCNN). After fully training and fine-tuning the three methods on the training set, the model parameters were obtained and evaluated on the validation set. The resulting performance metrics for airport runway detection and segmentation are shown in Table 4 and Table 5.

The results presented in Table 4 and Table 5 indicate that, after replacing the RPN network, the model utilizing the RRPN network exhibited significant improvements in both detection and segmentation accuracy for airport runways. The detection and segmentation accuracies of the R-Mask RCNN model on the airport runway dataset reached 72.28% and 66.51%, respectively, representing improvements of 4.89% and 6.91% compared to the Mask RCNN model. This indicates that the Mask RCNN network model, improved with rotated bounding boxes, effectively enhances the detection and segmentation accuracy of airport runway targets. On the other hand, the CR-Mask RCNN model, which further integrates an attention mechanism, achieved detection and segmentation accuracies of 77.07% and 73.59%, respectively, on the airport runway dataset, surpassing the Mask RCNN model by 9.68% and 13.99%. Compared to the R-Mask RCNN model, introducing the attention mechanism improved performance by 4.79% and 7.08%. This enhancement is attributed to the attention mechanism’s ability to strengthen the feature extractor’s response to runway targets, suppress background noise, and reduce false positives and false negatives, thereby improving the overall detection and segmentation accuracy of airport runways.

**Figure 10:**
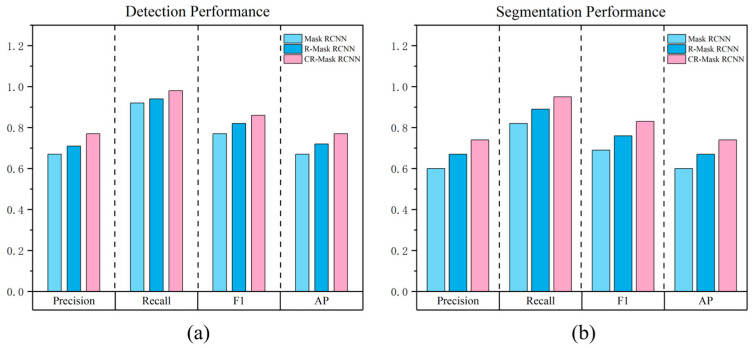
Comparison of detection and segmentation performance of different methods on airport runway dataset. (**a**) Detection performance; (**b**) segmentation performance.

To provide a more intuitive comparison of the performance of the three different remote sensing image-based airport runway detection and segmentation methods, Figure 10 presents histograms that compare the performance of these methods across each evaluation metric. The results clearly demonstrate that the method proposed in this study offers significant advantages in airport runway detection and segmentation using remote sensing imagery.

In Figure 10, the horizontal axis shows Precision, Recall, F1, and AP, representing four performance evaluation metrics. Each metric includes three remote sensing image airport runway detection and segmentation methods: Mask RCNN, R-Mask RCNN, and CR-Mask RCNN. The vertical axis represents the score of each method under different metrics. The four performance metrics of Precision, Recall, F1, and AP of the remote sensing image airport runway detection and segmentation method proposed in this paper outperform the other methods, indicating that the CR-Mask RCNN method has the best performance among the remote sensing image airport runway detection and segmentation methods.

To further illustrate the differences in the training efficiency of the different methods, Table 6 shows the time required for the training process of Mask RCNN, R-Mask RCNN, and CR-Mask RCNN. Although the improvement of rotated bounding boxes and the introduction of the attention mechanism provide higher detection and segmentation accuracy, their training time is longer, and this phenomenon is reflected in Table 6.

**Figure 11:**
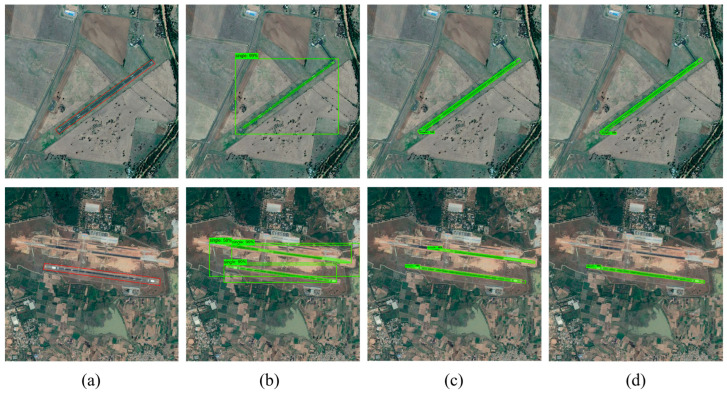
Comparison of detection and segmentation results for a single airport runway in small-scale images using different methods. (**a**) Original image, red boxes used to mark airport runways. (**b**) Detection and segmentation results using Mask RCNN. (**c**) Detection and segmentation results using R-Mask RCNN. (**d**) Detection and segmentation results using CR-Mask RCNN.

The experimental performance of the proposed method on the airport runway dataset is quite remarkable in terms of accuracy improvement, and the experimental results are verified numerically and visualized to present the excellent performance of this method in the runway detection task. The CR-Mask RCNN method used in this paper significantly outperforms the traditional method for detection on different test images and can segment airport runways and eliminate background interference effectively. Figure 11 shows the detection results of comparing different methods on small-scale images, from which it can be observed that CR-Mask RCNN achieves higher detection accuracy and more realistic segmentation in small-scale airport runway detection compared to other methods.

Figure 11a shows a portion of the original image selected from the airport runway validation dataset, with the red box indicating the airport runway area. The results reveal that, in the first image, due to the relatively simple background, all three methods successfully detected the single airport runway in the small-scale image with no false negatives or false positives. In the second image, however, the surroundings are more complex, and both Mask RCNN and R-Mask RCNN exhibit varying degrees of detection error, misclassifying surrounding roads as runway targets. Additionally, the prediction box generated by Mask RCNN is drawn as the largest enclosing rectangle of the runway, capturing significant irrelevant background information. In contrast, R-Mask RCNN and CR-Mask RCNN show substantial improvements in detection performance, with prediction boxes closely approximating the minimum enclosing rectangle of the runway, more accurately reflecting the actual shape and size of the runway. This removes unnecessary background information effectively, resulting in a more realistic estimation of the runway’s length, width and orientation. The detection results of CR-Mask RCNN align most closely with the actual contours and orientation of the runway, achieving optimal detection and segmentation accuracy and demonstrating a higher resolution of the runway structure.

**Figure 12:**
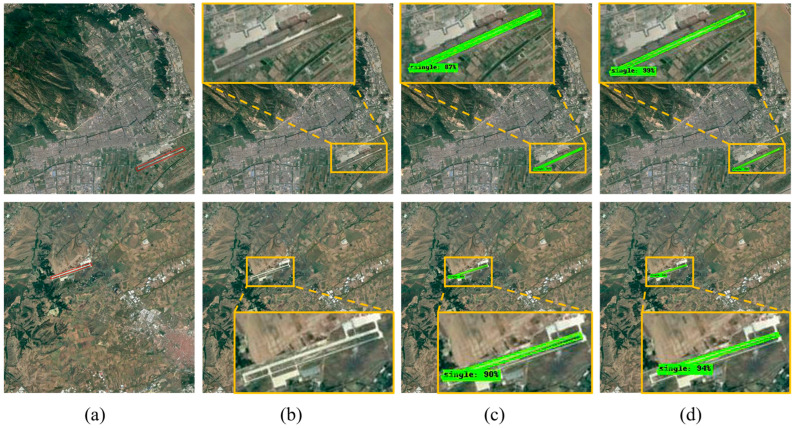
Comparison of detection and segmentation results for a single airport runway in large-scale images using different methods. (**a**) Original image, red boxes used to mark airport runways. (**b**) Detection and segmentation results using Mask RCNN. (**c**) Detection and segmentation results using R-Mask RCNN. (**d**) Detection and segmentation results using CR-Mask RCNN.

Furthermore, after achieving significant results in small-scale image detection and segmentation, this study further applies the method to airport runway detection and segmentation in large-scale images, and locally enlarges the results of airport runway detection and segmentation in the images, as shown in Figure 12.

The detection results indicate that, in large-scale images, Mask RCNN is limited in its ability to suppress background noise and is easily affected by complex features, often resulting in a high false negative rate, thereby failing to accurately detect the airport runway (Figure 12b). In contrast, R-Mask RCNN and CR-Mask RCNN demonstrate more vital generalization ability and higher detection accuracy in large-scale runway detection. R-Mask RCNN utilizes rotated bounding boxes to detect airport runways, significantly enhancing its ability to capture runway boundaries and allowing accurate runway outline recognition even in complex backgrounds, effectively reducing background noise interference (Figure 12c). Additionally, CR-Mask RCNN introduces an attention mechanism, further improving detection and segmentation precision. It performs pixel-level segmentation of the runway area, reducing false positives and negatives while precisely capturing edge details, resulting in segmentation outcomes that closely match the actual runway contours (Figure 12d).

**Figure 13:**
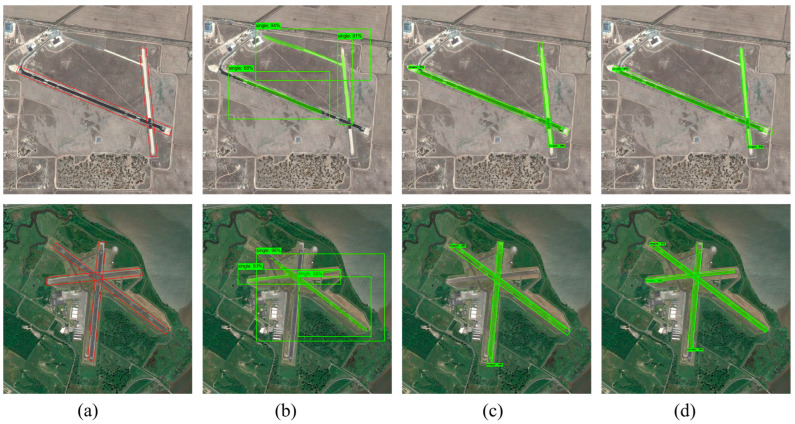
Comparison of detection and segmentation results for crossed airport runway in large-scale images using different methods. (**a**) Original image, red boxes used to mark airport runways. (**b**) Detection and segmentation results using Mask RCNN. (**c**) Detection and segmentation results using R-Mask RCNN. (**d**) Detection and segmentation results using CR-Mask RCNN.

Beyond the detection and segmentation experiments on single airport runways, this study further evaluates the applicability and accuracy of the three methods in scenarios involving multiple intersecting airport runways. Figure 13 compares the detection and segmentation results of different methods for multiple intersecting airport runway scenarios.

The results show that, in the first image, due to challenges such as small runway targets, narrow width, and similar features to roads in the remote sensing image, the Mask RCNN method mistakenly classifies roads as airport runways. In contrast, the R-Mask RCNN and CR-Mask RCNN methods accurately detect all runways without false positives. In the second image, there are multiple complex and intersecting airport runways. The three prediction boxes generated by Mask RCNN overlap significantly, creating a cluttered and difficult-to-interpret image. Mask RCNN and R-Mask RCNN exhibit false negatives. However, when the attention mechanism is introduced into the R-Mask RCNN model, all airport runways are accurately detected with no false negatives.

**Figure 14:**
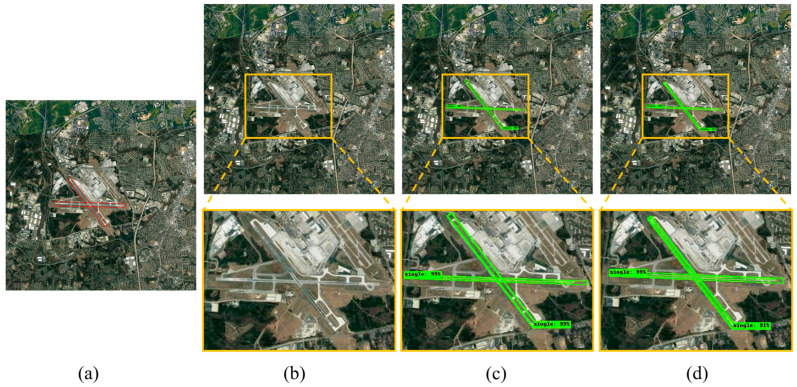
Comparison of detection and segmentation results for crossed airport runway in small-scale images using different methods. (**a**) Original image, red boxes used to mark airport runways. (**b**) Detection and segmentation results using Mask RCNN. (**c**) Detection and segmentation results using R-Mask RCNN. (**d**) Detection and segmentation results using CR-Mask RCNN.

Figure 14 further illustrates the detection and segmentation performance of different methods on intersecting airport runways in large-scale images, comparing the performances of each method in complex scenarios.

The results in Figure 14 demonstrate significant differences in the performance of the three methods for detecting intersecting airport runways in large-scale imagery. Mask RCNN struggles to accurately detect multiple intersecting airport runways in images with complex backgrounds (Figure 14b). In contrast, R-Mask RCNN shows improved accuracy in recognizing and extracting multiple intersecting runways from complex backgrounds, significantly enhancing detection performance; however, the segmentation detail still needs to be improved (Figure 14c). CR-Mask RCNN exhibits the best overall performance in both detection and segmentation. It not only precisely identifies the airport runway regions but also significantly improves the accuracy and completeness of the segmentation (Figure 14d).

The visual analysis of the airport runway detection results from the three methods reveals that the prediction box generated by Mask RCNN is drawn as the largest enclosing rectangle of the runway, containing a substantial amount of irrelevant background information. This leads to lower detection accuracy and positioning precision. Under these conditions, Mask RCNN exhibits a significantly higher false positive rate, demonstrating sensitivity to background noise and losing precise control over the runway target’s shape and edges in complex backgrounds. However, R-Mask RCNN captures airport runways’ shape and edge features more accurately than traditional Mask RCNN, showing clear advantages in detecting complex and intersecting runways. R-Mask RCNN enhances the edge detection capability, producing prediction boxes closer to the actual structure of the runway and effectively reducing background interference. However, R-Mask RCNN still experiences edge blurring and misclassification in complex environments. By comparison, CR-Mask RCNN, with an integrated attention mechanism, significantly improves detection and segmentation accuracy. CR-Mask RCNN more effectively suppresses false positives and false negatives in multi-target scenarios, with its detection and segmentation results aligning more closely with the actual runway in terms of shape, size, aspect ratio, and orientation. This dramatically enhances boundary precision and segmentation quality. Furthermore, CR-Mask RCNN reduces redundant false positives and false negatives by filtering out background noise, demonstrating superior resolution of runway structures and making it suitable for high-precision airport runway detection tasks.

## 4. Conclusions

This study proposed an improved Mask RCNN method for detecting and segmenting airport runways in remote sensing images. This method significantly improves the accuracy of airport runway detection and segmentation through integrating a rotated region proposal network (RRPN) and an attention mechanism into the Mask RCNN network model. The RRPN effectively solves the limitation of the traditional horizontal bounding box, which only considers the maximum outer rectangle of the target and reduces the interference from extraneous background information, thus enhancing the ability to capture airport runway features. In addition, an attention mechanism is further introduced to enhance the network’s ability to extract information from airport runway feature maps at different scales, strengthen the response to low-resolution but semantically rich feature maps, and appropriately regulate the response to high-resolution feature maps to improve the network’s ability to detect detailed information effectively. The experimental results show that the proposed method had significantly improved airport runway target detection and segmentation performance, when compared with the traditional method. After adopting the rotated bounding box method, the average precision (AP) was improved by 4.89% and 6.91% in airport runway detection and segmentation tasks, respectively. It was further improved by 4.79% and 7.08% after introducing the attention mechanism, highlighting the effectiveness of the proposed method. The method proposed in this paper is fully validated on images with different resolutions, and the results show that the method has strong generalization ability and good practicality. The experiments proved that the method provides an effective solution for the task of airport runway target detection and segmentation in remote sensing images.

Despite the advantages of the method proposed in this paper, there are some limitations. Although the rotated bounding box used in this paper effectively improves the detection and segmentation accuracy, it also brings a certain computational overhead, and the consumption of computational resources may become a bottleneck. Future research can aim to optimizing the computational efficiency of the rotated bounding box by introducing more efficient algorithms or hardware acceleration means to reduce the consumption of computational resources, so as to further enhance the practicality and scalability of the method.

## Figures and Tables

**Table 1:** Calculation steps of rotation IOU.

IOU Calculation Method
- Input all rotated rectangular boxes R_1_~R_j_;
- Select two rectangular boxes R_i_, R_j_;
- Put all the points (coordinates) that intersect both into the empty set H, which is then {e, f, g, h};
- Then put the vertex coordinates of R_i_ in R_j_ into H, which is then {e, f, g, h, a};
- The coordinates of the vertices of jR in iR are then put into H, which is then {e, f, g, h, a, D};
- Sort the set H in counterclockwise order as {a, h, D, g, f, e};
- Calculate the overlapping area Sc in H using triangulation;
- Finally yields IOU = S_c_/S_Ri_ + S_Rj_ − S_Rc_.

**Table 2:** Prediction results relationship matrix.

Real Situation	Classification Results	
	Airport Runway	Non-Airport Runway
Airport runway	TP	FN
Non-airport runway	FP	TN

**Table 3:** Statistical table of length and width ratio in airport runway dataset.

Aspect Ratio Range	Quantities	Volume (%)
1:1–1:10	25	2.32
1:10–1:20	48	4.45
1:20–1:30	237	21.99
1:30–1:40	354	32.84
1:40–1:50	263	24.40
1:50–1:60	104	9.65
1:60–1:70	34	3.15
1:70–1:80	8	0.74
1:80–1:90	3	0.28
1:90–1:100	2	0.19

**Table 4:** Comparison of detection results of three methods on airport runway dataset.

Model	Precision	Recall	F1	AP (%)
Mask RCNN	0.67	0.92	0.77	67.39
R-Mask RCNN	0.71	0.94	0.82	72.28
CR-Mask RCNN	0.77	0.98	0.86	77.07

**Table 5:** Comparison of segmentation results of three methods on airport runway dataset.

Model	Precision	Recall	F1	AP (%)
Mask RCNN	0.60	0.82	0.69	59.60
R-Mask RCNN	0.67	0.89	0.76	66.51
CR-Mask RCNN	0.74	0.95	0.83	73.59

**Table 6:** Evaluation table of training time for three methods.

Model	Training Time
Mask RCNN	3 h
R-Mask RCNN	4 h 30 min
CR-Mask RCNN	5 h 10 min

## Data Availability

The raw data supporting the conclusions of this article are unavailable due to privacy restrictions; further inquiries can be directed to the corresponding author.
